# Tuberculosis en niños y adolescentes en Ecuador: análisis de la notificación, las características de la enfermedad y el resultado del tratamiento

**DOI:** 10.26633/RPSP.2019.104

**Published:** 2019-12-20

**Authors:** Guido Silva, Freddy Pérez, Diana Marín

**Affiliations:** 1 Ministerio de Salud Pública de Ecuador Quito Ecuador Ministerio de Salud Pública de Ecuador, Quito, Ecuador.; 2 Organización Panamericana de la Salud Washington, D.C. Estados Unidos de América Organización Panamericana de la Salud, Washington, D.C., Estados Unidos de América.; 3 Universidad Pontificia Bolivariana Medellín Colombia Universidad Pontificia Bolivariana, Medellín, Colombia.

**Keywords:** Tuberculosis, resultado del tratamiento, niño, adolescente, Ecuador, Tuberculosis, treatment outcome, child, adolescent, Ecuador, Tuberculose, resultado do tratamento, criancas, adolescente, Ecuador

## Abstract

**Objetivos.:**

Estimar la carga de tuberculosis (TB) en menores de 15 años y describir las características clínico, epidemiológicas y los resultados del tratamiento antituberculoso en Ecuador.

**Métodos.:**

Se realizó un estudio retrospectivo utilizando los datos del programa nacional de TB de los años 2015 y 2016. Se estimaron la tasa y el porcentaje de casos de TB infantil y se describieron las características de la enfermedad y el resultado del tratamiento según las categorías de edad: 0-4, 5-9 y 10-14 años.

**Resultados.:**

De los 10 991 casos de TB diagnosticados, 223 (2,03%) fueron menores de 15 años; según la región del país esta carga varió entre 0 y 5,5%. De los 223 casos, en 213 se había registrado el resultado del tratamiento y fueron incluidos en el estudio; 78 (37%) eran menores de 5 años y en 147 (69%) no hubo registro de la investigación de contactos. Sesenta y cinco (68%) de los adolescentes y 40 (51%) de los menores de 5 años tenían diagnóstico de TB pulmonar. La prevalencia de VIH fue 11,5% en los menores 5 años y 6,3% en el grupo de 10-14 años. El tratamiento fue satisfactorio en el 93% de los casos, (curación, 36,6%, tratamiento terminado, 56,8%).

**Conclusiones:**

Ecuador presenta un alto porcentaje de subdiagnóstico de TB infantil y una carga menor a la esperada, principalmente en menores de 5 años. La alta prevalencia de VIH y la falta de sistematización adecuada de la investigación de contactos en los adolescentes revelan la necesidad de considerar estrategias centradas en la familia y que involucren la capacitación del personal de salud en el manejo del paciente pediátrico centrándose en las necesidades específicas de cada población.

## Abstract (en)

**Objectives.:**

To estimate the tuberculosis (TB) burden in children under 15 years of age and to describe the clinical and epidemiological characteristics and the results of the anti-tuberculosis treatment in Ecuador.

**Methods.:**

A retrospective study was carried out using data from the national TB programm for 2015 and 2016. The rate and percentage of cases of childhood TB were estimated and the disease characteristics and treatment outcome were described according to age categories: 0-4, 5-9 and 10-14 years.

**Results.:**

Of the 10 991 cases of TB diagnosed, 223 (2.03%) were under 15 years of age; depending on the region, this burden ranged from 0 to 5.5%. Of the 223 cases, 213 had their treatment outcome registered and were included in the study; 78 (37%) were younger than 5 years and 147 (69%) had no record of contact screening. Sixty-five (68%) of the adolescents and 40 (51%) of the children under 5 had a diagnosis of pulmonary TB. HIV prevalence was 11.5% in children under 5 and 6.3% in the 10-14 age group. Treatment was succesful in 93% of cases (cure, 36.6%, treatment completed, 56.8%).

**Conclusions.:**

Ecuador presents a high percentage of under-diagnosis of childhood TB and a lower than expected burden, mainly in children under 5 years of age. The high prevalence of HIV and the lack of adequate systematization of adolescent contact screening suggest the need to consider family-centered strategies that involve training health personnel in the management of pediatric patients, with a focus on the specific needs of each population.

## RESUMO

**Objetivos.:**

Estimar a carga de tuberculose (TB) em crianças menores de 15 anos e descrever as características clínicas e epidemiológicas e os resultados do tratamento antituberculose no Equador.

**Métodos.:**

Foi realizado um estudo retrospectivo utilizando dados do programa nacional de TB para os anos de 2015 e 2016. A taxa e a porcentagem de casos de tuberculose infantil foram estimadas. As características da doença e o resultado do tratamento foram descritos de acordo com as categorias de idade: 0 a 4 anos, 5 a 9 anos e 10 a 14 anos.

**Resultados.:**

Dos 10 991 casos de TB diagnosticados, 223 (2,03%) tinham menos de 15 anos. A carga de TB variou de 0 a 5,5%, dependendo da região do país. Em 213 dos 223 casos, o resultado do tratamento havia sido registrado, possibilitando a inclusão no estudo. Desses, 78 (37%) tinham menos de 5 anos; em 147 (69%) não havia registro da investigação de contato. Sessenta e cinco (68%) dos adolescentes e 40 (51%) dos menores de 5 anos foram diagnosticados com TB pulmonar. A prevalência de HIV foi de 11,5% em crianças menores de 5 anos e de 6,3% no grupo de 10 a 14 anos. O tratamento foi satisfatório em 93% dos casos (cura, 36,6%; tratamento concluído, 56,8%).

**Conclusões.:**

O Equador tem uma elevada porcentagem de subdiagnóstico de TB infantil e a carga da doença ficou abaixo do esperado, principalmente em crianças menores de 5 anos. A alta prevalência do HIV e a falta de sistematização da pesquisa de contatos em adolescentes revelam a necessidade de considerar estratégias centradas na família, que envolvam o treinamento de profissionais de saúde no manejo do paciente pediátrico, com foco nas necessidades específicas de cada população.

En 2017, en todo el mundo se registraron más de 10 millones de pacientes nuevos con tuberculosis (TB), de los cuales un millón fueron menores de 15 años. De las 233 000 muertes por TB en menores de 15 años el 80% ocurrió en menores de 5 años y el 96% en niños que no habían accedido a tratamiento (1). En 2014, la Organización Mundial de la Salud (OMS) lanzó la Estrategia Fin a la TB. Entre sus objetivos, previstos para el 2035, se incluye la reducción del 90% en la incidencia de TB en comparación con 2015 (2), pero el logro de este objetivo puede verse comprometido por la falta de datos de referencia nacionales sólidos en la población infantil y por la poca prioridad asignada a los programas de control de la TB (3).

A ello se suman otros factores, como el reporte deficiente de casos, que imposibilitan estimar con precisión la carga global de la TB en la población infantil. Las mejores (4) estimaciones sugieren que la población menor de 15 años debería corresponder a cerca del 10% de toda la carga de la enfermedad por tuberculosis, sugiriendo que anualmente cerca de 32 000 casos de TB infantil no son diagnosticados y por ende no reportados (5).

Entre los principales obstáculos identificados para alcanzar las metas de la Estrategia Fin a la TB en esta población están la limitada implementación de investigación de casos y de acceso a la terapia preventiva, la dificultad en el diagnóstico (que en ocasiones se hace en hospitales de referencia, o simplemente no son diagnosticados) y la falta de reporte del resultado del tratamiento antituberculoso discriminado por niños y adolescentes (6). Asimismo, existe una falta de capacidad instalada que garantice el conocimiento y la confianza de los trabajadores de la salud en la prevención, el diagnóstico y el manejo de niños y adolescentes expuestos a la TB.

Se considera que Ecuador ocupa el noveno lugar en la Región de las Américas en lo que respecta a la carga de TB. La tasa de incidencia notificada en el 2017 fue 43 casos nuevos por 100 000 habitantes y, como otros países, no cuenta con datos de la magnitud de esta enfermedad en niños y adolescentes. Por lo tanto, no ha sido posible establecer objetivos para la TB infantil y direccionar estrategias para lograrlos (5-8).

Con base en lo anterior, la limitada investigación en niños y adolescentes con TB en la Región y el llamado de la OMS a la realización de investigación operativa e investigación de los sistemas y servicios de salud (9-12), los objetivos de este artículo son estimar la carga de TB infantil general y según cada coordinación zonal de salud, describir las características clínicas y epidemiológicas de niños y adolescentes y presentar los resultados del tratamiento antituberculoso en esta población en Ecuador.

## MATERIALES Y MÉTODOS

Se realizó una investigación operativa con diseño de cohorte retrospectiva que evaluó el perfil de la tuberculosis infantil en Ecuador a partir de los datos recolectados rutinariamente en el programa nacional.

Ecuador, en América Latina, posee actualmente 16,6 millones de habitantes; de ellos, el 31% son menores de 15 años. Está organizado en nueve zonas administrativas que agrupan a las 24 provincias y a los distintos distritos que facilitan la vigilancia en salud pública.

Con relación al diagnóstico de TB, los casos son reportados a la Estrategia Nacional de Prevención y Control de Tuberculosis (ENPCTB) por el personal médico a cargo del establecimiento de salud de cada distrito. Desde 2015, el Ministerio de Salud Pública ha implementado gradualmente el acceso universal a GeneXpert MTB/RIF (Cepheid, Sunnyvale, CA, USA); a la prueba cutánea de tuberculina solo tiene acceso la población con seguro de salud privado (15%). La investigación y seguimiento de contactos es obligatoria para todos los miembros del hogar y los contactos cercanos del caso índice fuera del hogar. Los contactos menores de 5 años, independiente de los síntomas respiratorios, son evaluados por el pediatra y el médico acreditado de TB en el establecimiento de salud. El diagnóstico de casos pediátricos está basado en el criterio clínico, epidemiológico (identificación de contacto con TB), bacteriológico (baciloscopia, cultivo) y radiológico (rayos X). Para el diagnóstico bacteriológico se toman dos muestras diarias por aspirado gástrico, esputo inducido y, en casos excepcionales, una muestra diaria por lavado bronquioalveolar. El diagnóstico y el tratamiento para la TB sensible y resistente son gratuitos para los pacientes; el tratamiento se administra en los centros de salud que brindan tratamiento directamente observado a todos los pacientes. El esquema terapéutico es estándar y se siguen las recomendaciones de la OMS (7,13,14).

La población en estudio consistió en todos los casos notificados en menores de 15 años que habían sido diagnosticados por medios bacteriológicos o clínicos y que iniciaron tratamiento para tuberculosis sensible entre el 1 de enero de 2015 y el 31 de diciembre de 2016. Se excluyeron aquellos sin resultado en el tratamiento al 31 de diciembre de 2016 y con diagnóstico de tuberculosis resistente.

Los datos fueron obtenidos de los informes trimestrales enviados al ENPCTB por las coordinaciones zonales que a su vez los reciben de los centros de salud, y las proyecciones de población realizadas por el Instituto de Estadísticas y Censos del Ecuador (INEC)

Los datos de interés fueron el total de casos de TB, los casos de TB en menores de 15 años y el centro de salud que reportó para identificar la coordinación zonal, ya que en Ecuador el Ministerio de Salud Pública organizó coordinaciones zonales de salud de intervención que abarcan provincias y distritos político-administrativos con el objetivo de efectuar intervenciones rápidas en problemas de salud pública.

Otra fuente de datos fueron las tarjetas de tratamiento de los pacientes que reposan en los centros de salud, donde reciben atención y tratamiento para la TB. De estas tarjetas, las variables de interés fueron la edad en años, el sexo (masculino, femenino), el número de contactos con TB (1 a 2, 3 o más contactos), la condición de ingreso (nuevo, recaída, tratamiento después del fracaso), la forma clínica (pulmonar, extrapulmonar), el tipo de diagnóstico (bacteriológico, clínico, rayos x), la coinfección TB/VIH (sí, no) y el resultado del tratamiento antituberculoso (curado, tratamiento completo, fracaso del tratamiento, pérdida en el seguimiento y fallecido) según las definiciones de la OMS. Se consideró tratamiento exitoso a la suma de pacientes curados y tratamiento completos (15). Para algunos análisis la edad se agrupó en menores de 1 año, 1-4, 5-9 y 10-14 años.

Para el análisis se estimó la tasa de incidencia anual de TB total e infantil multiplicadas por 100 000 habitantes y también el porcentaje de casos de TB infantil, tomando como denominador el total de casos de TB notificados en cada coordinación zonal de salud. Se realizó la georreferenciación de las tasas de TB en menores de 15 años por provincia administrativa utilizando los programas Tableau 2019.2 y ArcGis 9.2 (16-19)

Se describieron las características sociodemográficas y clínicas de los casos según el grupo de edad (de 0-4, 5-9 y 10-14 años) y se analizaron a través de la prueba Chi cuadrado las posibles diferencias en los porcentajes de egreso del tratamiento, según las características sociodemográficas y clínicas de los casos. Se consideraron diferencias significativas con valor *p* bilateral < 0,05. Los análisis se realizaron en los programas Epi Info 7.0 y en Stata 14.0.

Se recibió aprobación por escrito de la Dirección Nacional de Estrategias de Prevención y Control del Ministerio de Salud de Ecuador para utilizar los datos de la base de datos nacional y acceder a las tarjetas de tratamiento de los años 2015 y 2016. El protocolo fue aprobado por el Comité de Ética del Hospital Eugenio Espejo de Quito (Ecuador) y el Comité de Revisión de Ética de la Organización Panamericana de la Salud (PAHOERC); este último concedió la exoneración del consentimiento informado de los participantes. La investigación se clasificó sin riesgo para los participantes debido a que la población de estudio ya había culminado el tratamiento para la tuberculosis y, por lo tanto, no era posible acceder directamente a ellos. Los datos por parte del PNCT fueron entregados y manejados con códigos sin número de identificación, nombres ni dirección de residencia a fin de garantizar el anonimato de los participantes.

**CUADRO 1.** Tasa de incidencia anual de tuberculosis por 100 000 habitantes y proporción de TB infantil según distribución de las Coordinaciones Zonales de Salud. Ecuador, 2015-2016

Coordinación Zonal ^a^	Casos TB total	Casos TB infantil	Población total	Población infantil	% TB infantil ^b^	Tasa TB general	Tasa TB infantil
Zona 1	623	15	2 770 581	967 679	2,41	22,49	1,55
Zona 2.	289	16	1 042 888	464 825	5,54	27,71	3,44
Zona 3	455	25	3 148 495	1 008 953	5,49	14,45	2,48
Zona 4	934	12	3 737 011	1 205 239	1,28	24,99	1,00
Zona 5	1 893	16	4 804 552	1 704 970	0,85	39,40	0,94
Zona 6	343	0	2 395 597	788 679	0,00	14,32	0,00
Zona 7	818	6	2 479 660	782 473	0,73	32,99	0,77
Zona 8	5 226	126	5 943 103	1 666 677	2,41	87,93	7,56
Zona 9	410	7	5 149 710	1 389 760	1,71	7,96	0,50
**Total**	**10 991**	**223**	**31 471 597**	**9 979 255**	**2,03**	**34,92**	**2,23**

**Fuente:** elaboración propia a partir de los resultados presentados

a Zona 1: Provincias de Esmeraldas, Imbabura, Carchi y Sucumbíos.; Zona 2: Provincias de Pichincha, Napo Orellana excepto cantón Quito.; Zona 3: Provincias de Cotopaxi, Tungurahua, Chimborazo, Pastaza; Zona 4: Provincias de Manabí, Santo Domingo de los Tsáchilas.; Zona 5: Provincias de Santa Elena Guayas, Los Ríos, Santa Elena y Galápagos excepto cantón Guayaquil, Samborondón y Durán.; Zona 6: Provincias de Cañar, Azuay, Morona Santiago.; Zona 7: Provincias de El Oro, Loja y Zamora Chinchipe.; Zona 8: Cantón Guayaquil, Samborondón y Durán.; Zona 9: Cantón Quito

b Corresponde a la relación: (Casos de TB infantil / Casos de TB total) *100

## RESULTADOS

**FIGURA 1.**
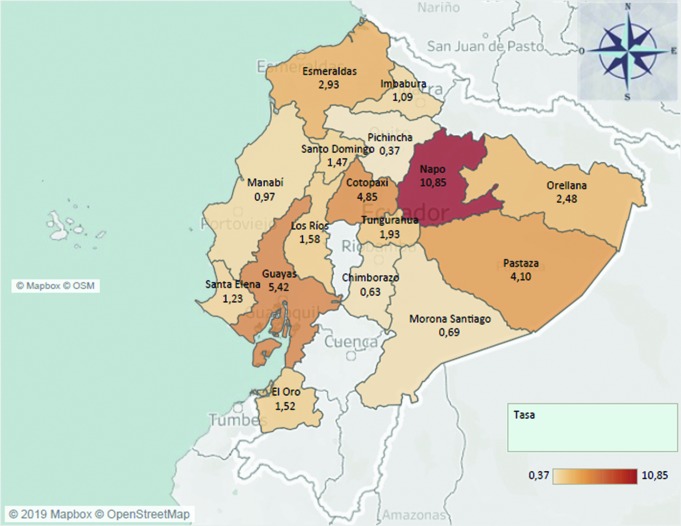
Distribución de la tasa de tuberculosis infantil por división política o provincias. Ecuador, 2015-2016.

Entre 2015 y 2016 se diagnosticaron 10 991 casos de TB, lo que representa una tasa de 34,92 por 100 000 habitantes. Al analizar la incidencia de TB infantil por coordinación zonal de salud se encontró que fue mayor en la Zona 8 (7,56 por 100 000), donde también se concentró la mayor tasa de TB en el país (87,9 por 100 000). Sin embargo, al analizar por provincia administrativa se encontró que la mayor incidencia de TB infantil se ubica en Napo, donde alcanza hasta 10,35 casos por 100 000. La proporción de casos en menores de 15 años en el país fue 223 casos (2,03%) pero varió entre 0% y 5,54%. (Cuadro 1, Figura 1).

**FIGURA 2.**
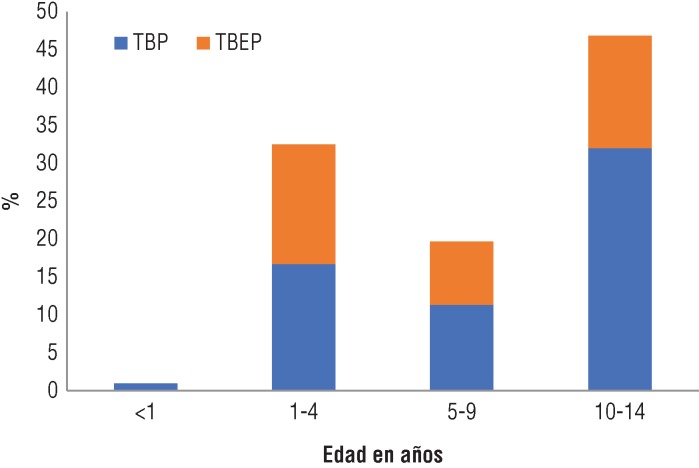
Distribución porcentual de los casos de TB según la forma clínica y la edad. Ecuador, 2015-2016

La forma clínica de la enfermedad en relación con la de edad tuvo una variación significativa respecto de lo esperado. En menores de 1 año se presentaron 2 casos (1%) de TB pulmonar, mientras que, en adolescentes de 10 a 14 años, 65 casos (32%). No se encontró ningún caso de TB extrapulmonar en los menores de 1 año (Figura 2).

De los 223 casos diagnosticados, 6 fueron excluidos por no tener resultado de su tratamiento antituberculoso y 4 por tener diagnóstico de TB resistente que, por requerir tratamiento prolongado, el resultado de su tratamiento no estaba disponible al cerrar los años de estudio. Entre los 213 casos incluidos en el estudio se encontró que 78 (37%) eran menores de 5 años y que 104 (49%) eran mujeres. En 147 (69%) de los casos no se encontró información registrada sobre el contacto de pacientes con TB y esto fue más frecuente en la población de 10 a 14 años. El criterio diagnóstico fue variable por grupo de edad; en los adolescentes de 10-14 años predominó el criterio bacteriológico. El porcentaje global de coinfección TB/VIH fue 9% (20 casos), los cuales fueron valores más bajos en adolescentes a diferencia de otras edades (11,5% vs 6,3%) (Cuadro 2).

En 199 casos (93,4%) el tratamiento fue exitoso, y entre quienes no tuvieron un resultado exitoso, 3 casos se clasificaron como fracaso del tratamiento, 6 tuvieron pérdida en el seguimiento y 5 fallecieron, 4 de los cuales eran menores de 5 años. Los 20 casos con coinfección TB/VIH egresaron con tratamiento exitoso. Se encontraron diferencias en los egresos según edad, forma clínica y método diagnóstico (Cuadro 3).

## DISCUSIÓN

Este es el primer estudio realizado en Ecuador que presenta la descripción de las características clínicas, epidemiológicas y los resultados del tratamiento de niños y adolescentes con TB sensible. Este estudio mostró un alto porcentaje de subdiagnóstico de TB infantil, una alta prevalencia de VIH –en especial en los menores de 10 años– y el cumplimiento de la meta de la OMS de tratamiento exitoso del 90% en la población infantil diagnosticada.

La afirmación un alto porcentaje de subdiagnóstico de TB infantil se basa en que aproximadamente el 10% de todos los casos de TB son niños (5) y más de la mitad ocurre en menores de 5 años (1). En este estudio la cantidad de casos reportados de TB infantil (2,03%) fue inferior a la esperada para la Región (12%) y a la reportada en los 22 países con mayor carga de la enfermedad a nivel mundial (9,6%) (4,20). Asimismo, aunque se espera que en los niños más pequeños entre el 10 y 20% de los casos consistan en tuberculosis pulmonar diseminada en Ecuador no se registró ninguno, lo cual es consistente con el subregistro y subreporte de 69% estimado por la OMS en este grupo etario (6,21,22). El diagnóstico de la TB infantil continúa siendo un desafío, y según un estudio realizado en Perú por Chiang y cols. estos resultados podrían deberse a las barreras que impiden realizar un diagnóstico óptimo (23). En ese estudio, las principales barreras encontradas fueron la inadecuada capacitación del personal de salud, el acceso limitado a las pruebas diagnósticas y la insuficiente investigación de los contactos. En el presente estudio no fue posible identificar si los casos fueron diagnosticados por medio de la búsqueda de contactos o de la consulta médica dado que esta información no se registra rutinariamente en las estrategias de prevención y control de los centros de salud. Es esperable que muchos de los casos hayan tenido contacto con un adulto con TB (4,21) y esto se reportó en el 31% de los casos. No obstante, el alto porcentaje de casos –y, en especial, en adolescentes (76,8%)– en quienes se desconoce esta información probablemente indica que no se está capturando la detección de contactos más allá de las estrategias de prevención y control de los centros de salud o que existe una limitación en la investigación de todos los contactos domiciliarios, aun cuando esto es obligatorio en Ecuador y en el mundo (6).

**Fuente:** elaboración propia a partir de los resultados presentados

Leyenda: Mapa del Ecuador dividido en sus 24 provincias oficiales, coloreadas según la tasa de TB en menores de 15 años. A mayor intensidad de color, mayor tasa de incidencia de TB. Las fronteras y nombres que se incluyen en este mapa tienen el propósito exclusivo de ilustración, y no implican juicio alguno, ni aval o aceptación por parte de la Revista Panamericana de Salud Pública o la Organización Panamericana de la Salud.

TBP: Tuberculosis pulmonar. TBEP: Tuberculosis extrapulmonar.

**Fuente:** elaboración propia a partir de los resultados presentados

En Ecuador el diagnóstico se basa principalmente en la evaluación clínica, una radiografía sugestiva de TB y, en los casos en que es factible, el diagnóstico bacteriológico (21). El uso de GeneXpert MTB/RIF (Cepheid, Sunnyvale, CA, Estados Unidos) –recomendado como prioritario por la OMS para el diagnóstico de la TB en la población infantil– fue implementado en Ecuador desde el 2015 pero solo desde 2017 la población infantil fue priorizada por la ENPCTB. Esto puede explicar porque en este estudio se efectuó el diagnóstico mediante GeneXpert MTB/RIFúnicamente en 4 casos (1,9%). La prueba cutánea de la tuberculina tampoco se utilizó como método diagnóstico debido a que generalmente no se encuentra disponible en el sector público en Ecuador (24). Adicionalmente, no se contó con información sobre el estado de vacunación con el bacilo de Calmette-Guérin (BCG) dado que programáticamente no se recolecta esta información. La cobertura con BCG en el Ecuador ha disminuido desde el 2010 hasta una cobertura del 83% en el 2016 (25), un nivel subóptimo en comparación con el resto del mundo (26). En Ecuador se requieren estrategias para mejorar la cobertura de vacunación al nacimiento y estudios que evalúen programáticamente el diagnóstico y el manejo de la TB infantil en el primer y segundo nivel de atención, donde se han demostrado oportunidades de prevención (27).

**CUADRO 2.** Características sociodemográficas y clínicas en la población infantil que inició tratamiento para la tuberculosis. Ecuador, 2015-2016

Característica	Grupo de edad			Total
	0 - 4	5 - 9	10 -14	N = 213
	N = 78	N = 40	N = 95	
**Sexo, n (%)**				
Masculino	39 (50,0)	20 (50,0)	50 (52,6)	109 (51,2)
Femenino	39 (50,0)	20 (50,0)	45 (47,4)	104 (48.8)
**Contactos con TB, n (%)**				
No registrado	45 (57,7)	29 (72,5)	73 (76,8)	147 (69,0)
1 – 2	26 (33,3)	8 (20,0)	13 (13,7)	47 (22,1)
3 o más	7 (9,0)	3 (7,5)	9 (9,5)	19 (8,9)
**Condición al ingreso, n (%)**				
Nuevo	76 (97,4)	39 (97,5)	93 (97,9)	208 (97,7)
Tto. después del fracaso	1 (1,3)	0 (0,0)	0 (0,0)	1 (0,5)
Recaída	1 (1,3)	1 (2,5)	2 (2,1)	4 (1,9)
**Forma clínica, n (%)**				
TB pulmonar	40 (51,3)	23 (57,5)	65 (68,4)	128 (60,1)
TB extrapulmonar	38 (48,7)	17 (42,5)	30 (31,6)	85 (39,9)
**Diagnóstico, n (%)**				
Sin datos	9 (11,5)	6 (15,0)	10 (10,5)	25 (11,7)
Bacteriológico	12 (15,4)	11 (27,5)	35 (36,8)	58 (27,2)
Clínica	7 (9,0)	6 (15,0)	11 (11,6)	24 (11,3)
Rayos X	23 (29,5)	6 (15,0)	19 (20,0)	48 (22,5)
Clínica y rayos X	27 (34,6)	11 (27,5)	20 (21,1)	58 (27,2)
**Coinfección TB/VIH, n (%)**				
Sí	9 (11,5)	5 (12, 5)	6 (6,3)	20 (9,4)

**Fuente:** elaboración propia a partir de los resultados presentados

Tto.: tratamiento; TB: tuberculosis.

Otro resultado importante en este estudio fue la prevalencia de VIH en los tres grupos de edad, similar a la de países con prevalencia moderada de VIH, que según la OMS varía entre 10 y 60% (28). En la Región, un estudio en Brasil encontró una prevalencia de VIH de 17% en población infantil con diagnóstico de TB (28), mientras que en otro estudio en Cuba la prevalencia fue 0% (29). Este último resultado es llamativo en comparación con el del presente estudio (9,4%) y cuando se analiza la prevalencia de VIH en adultos en Ecuador (0,3%) y Cuba (0,4%). Una posible explicación de este resultado es el pobre desempeño de Ecuador en comparación con Cuba respecto de las metas de eliminación de la transmisión materno infantil (30), y que la terapia preventiva de la TB en población infantil con VIH aunque reduce sustancialmente el riesgo de TB y está recomendada como parte de la atención integral del VIH (31), no se reporta en ninguno de los casos que hicieron parte de otro estudio en Ecuador (32).

Este es el primer estudio en reportar las características epidemiológicas y el resultado del tratamiento en adolescentes de 10 a 14 años con diagnóstico de TB. En este grupo la enfermedad se caracteriza por ser predominantemente pulmonar (6,33) y esto podría incrementar la oportunidad de propagación en entornos escolares hasta 21 veces más (OR: 22,5, IC95%: 5,9-191,4) (34). El éxito en el tratamiento es superior a lo esperado, con bajo porcentaje de pérdidas en el seguimiento, que podría mejorarse desarrollando servicios de salud para adolescentes que incluyan apoyo psicosocial y una interrupción mínima de la educación.

Respecto a la confirmación bacteriológica de la TB infantil, en este estudio se encontró el porcentaje de tratamiento exitoso más alto de la Región, un dato consistente con los reportados en países con alta carga de tuberculosis (31-34). Si bien el porcentaje de tratamiento no exitoso fue 6,6%, se encontró que de los 5 casos que murieron 4 tenían menos de 5 años. De ellos, 3 no habían alcanzado el primer año y 1 había ingresado por recaída.

Este estudio tiene varias limitaciones: 1) se utilizaron las tarjetas de tratamiento como principal fuente de información, y estas tenían datos incompletos; 2) los potenciales confusores –como el tipo de seguro de salud, la presencia de cicatriz de la vacuna BCG, las comorbilidades y el antecedente de tratamiento preventivo para la TB– no fueron medidos porque el estudio tuvo un diseño retrospectivo; 3) en el análisis de los resultados del tratamiento se excluyeron los casos que no habían iniciado tratamiento y esto pudo haber conducido a un sesgo de información para conocer las características de todos los niños y adolescentes que fueron diagnosticados con TB sensible.

**CUADRO 3.** Resultado del tratamiento para la TB según características epidemiológicas y clínicas de la población infantil. Ecuador, 2015-2016

Característica	Curado	Tratamiento completo	Fracaso del tratamiento	Pérdida en el seguimiento	Fallecido	Total N= 213	p
General	N = 78 (36,6%)	N = 121 (56,8%)	N = 3 (1,4%)	N = 6 (2,8%)	N = 5 (2,3%)		
**Edad (años), %**							
0-4	15,4	78,2	1,3	0,0	5,1	78	<0,001
5-9	32,5	65,0	0,0	2,5	0,0	40	
10-14	55,8	35,8	2,1	5,3	1,1	95	
**Sexo, %**							
Femenino	34,0	58,7	0,9	4,6	1,8	109	0,449
Masculino	39,4	54,8	1,9	1,0	2,9	104	
**Contactos con TB, %**							
Sin datos	42,1	51,7	1,4	1,4	3,4	147	0,112
1-2	21,3	70,2	2,1	6,4	0,0	47	
3 o más	31,6	63,1	0,0	5,3	0,0	19	
**Condición al ingreso, %**							
Nuevo	36,1	57,7	1,4	2,9	1,9	208	0,162
Tratamiento después del fracaso	100,0	0,0	0,0	0,0	0,0	1	
Recaída	50,0	25,0	0,0	0,0	25,0	4	
**Forma clínica, %**							
TB pulmonar	53,1	39,0	1,6	4,7	1,6	128	<0,001
TB extrapulmonar	0	95,3	1,2	0,0	3,5	85	
**Diagnóstico, %**							
Sin datos	8,0	88,0	0,0	0,0	4,0	25	<0,001
Bacteriológico	60,4	29,4	3,4	3,4	3,4	58	
Clínica	29,2	62,5	0,0	8,3	0,0	24	
Clínica y rayos X	32,1	63,2	0,9	1,9	1,9	106	
**Coinfección TB/VIH, %**							
No	38,3	54,4	1,6	3,1	2,6	193	0,267
Sí	20,0	80,0	0,0	0,0	0,0	20	

**Fuente:** elaboración propia a partir de los resultados presentados

Los resultados de este estudio destacan la necesidad de que las autoridades nacionales de salud de nivel central y descentralizado, así como otros actores que brindan servicios de salud para el manejo de la TB, implementen estrategias basadas en el ciclo de vida, inclusive intervenciones integrales centradas en la familia y en la comunidad. Es necesario un enfoque específico para la prevención y el tratamiento de la TB en los adolescentes, e incorporar pruebas diagnósticas que permitan un diagnóstico oportuno de la TB en los niños y adolescentes. Se requiere mayor capacidad instalada para garantizar el conocimiento y la confianza de los trabajadores de la salud del primer y segundo nivel para la prevención, el diagnóstico y el manejo de niños y adolescentes expuestos a la TB.

En conclusión, es evidente que existe un bajo diagnóstico de la tuberculosis en la infancia pues la carga comparada con el total de casos es menor que lo esperado, especialmente en los menores de 5 años. Aunque no es posible asegurar que esto se deba a una baja búsqueda de contactos, en la mayoría de los casos del estudio no existe registro del contacto inicial. A ello se suma una alta prevalencia de VIH, todo lo que implica la necesidad de considerar una mayor capacitación del personal. El éxito del tratamiento fue muy elevado –inclusive los casos curados como los tratamientos culminados– lo que posiblemente se debe a la protección y tutoría paternal hacia el niño.

Se sugiere fortalecer e implementar nuevas estrategias de captación, búsqueda de contactos, casos presuntivos de TB y manejo de los pacientes pediátricos en los centros de salud del país; incorporar pruebas diagnósticas (PPD, IGRAS, radiología, tomografía, pruebas moleculares); aumentar los recursos humanos capacitados en el primer y segundo nivel de atención para la toma adecuada de muestras y el manejo y el tratamiento de los pacientes pediátricos.

## Contribución de los autores.

GS, FP y DM concibieron el estudio original, planificaron los experimentos, recolectaron y analizaron los datos, interpretaron los resultados, escribieron y revisaron el manuscrito. Todos los autores han leído y aprobado el manuscrito y han contribuido significativamente al trabajo.

## Agradecimientos.

Esta investigación se llevó a cabo mediante la Iniciativa de Capacitación Estructurada en Investigación Operativa (SORT IT, por sus siglas en inglés), una alianza mundial dirigida por el Programa Especial de Investigación y Capacitación de Enfermedades Tropicales de la Organización Mundial de la Salud (OMS/TDR) y el Departamento de Enfermedades Transmisibles y Determinantes Ambientales de la Salud de la Organización Panamericana de la Salud. Se obtuvo financiamiento de la oficina subregional andina de la Organización Panamericana de la Salud. Los financiadores no desempeñaron ningún papel en el diseño del estudio, la recopilación y el análisis de los datos, la decisión de publicar ni la elaboración del artículo.

## Declaración.

Las opiniones expresadas en este manuscrito son responsabilidad del autor y no reflejan necesariamente los criterios ni la política de la *RPSP/PAJPH* o de la OPS.
